# Extraction of Roads Using the Archimedes Tuning Process with the Quantum Dilated Convolutional Neural Network

**DOI:** 10.3390/s23218783

**Published:** 2023-10-28

**Authors:** Mohd Jawed Khan, Pankaj Pratap Singh, Biswajeet Pradhan, Abdullah Alamri, Chang-Wook Lee

**Affiliations:** 1 Department of Computer Science & Engineering, Central Institute of Technology, Kokrajhar 783370, Assam, India; pankajp.singh@cit.ac.in; 2 Centre for Advanced Modelling and Geospatial Information Systems, School of Civil and Environmental Engineering, University of Technology Sydney, Sydney 2007, Australia; biswajeet.pradhan@uts.edu.au; 3 Institute of Climate Change, Universiti Kebangsaan Malaysia, Bangi 43600, Malaysia; 4 Department of Geology and Geophysics, College of Science, King Saud University, Riyadh 21589, Saudi Arabia; amsamri@ksu.edu.sa; 5 Department of Science Education, Kangwon National University, 1 Gangwondaehak-gil, Chuncheon-si 24341, Republic of Korea

**Keywords:** road extraction, remote sensing, convolutional neural networks, dilated convolutions, quantum computing, Archimedes optimization algorithm, artificial intelligence

## Abstract

Road network extraction is a significant challenge in remote sensing (RS). Automated techniques for interpreting RS imagery offer a cost-effective solution for obtaining road network data quickly, surpassing traditional visual interpretation methods. However, the diverse characteristics of road networks, such as varying lengths, widths, materials, and geometries across different regions, pose a formidable obstacle for road extraction from RS imagery. The issue of road extraction can be defined as a task that involves capturing contextual and complex elements while also preserving boundary information and producing high-resolution road segmentation maps for RS data. The objective of the proposed Archimedes tuning process quantum dilated convolutional neural network for road Extraction (ATP-QDCNNRE) technology is to tackle the aforementioned issues by enhancing the efficacy of image segmentation outcomes that exploit remote sensing imagery, coupled with Archimedes optimization algorithm methods (AOA). The findings of this study demonstrate the enhanced road-extraction capabilities achieved by the ATP-QDCNNRE method when used with remote sensing imagery. The ATP-QDCNNRE method employs DL and a hyperparameter tuning process to generate high-resolution road segmentation maps. The basis of this approach lies in the QDCNN model, which incorporates quantum computing (QC) concepts and dilated convolutions to enhance the network’s ability to capture both local and global contextual information. Dilated convolutions also enhance the receptive field while maintaining spatial resolution, allowing fine road features to be extracted. ATP-based hyperparameter modifications improve QDCNNRE road extraction. To evaluate the effectiveness of the ATP-QDCNNRE system, benchmark databases are used to assess its simulation results. The experimental results show that ATP-QDCNNRE performed with an intersection over union (IoU) of 75.28%, mean intersection over union (MIoU) of 95.19%, F1 of 90.85%, precision of 87.54%, and recall of 94.41% in the Massachusetts road dataset. These findings demonstrate the superior efficiency of this technique compared to more recent methods.

## 1. Introduction

Remote sensing images (RSI) find diverse applications in urban planning, building footprints extraction, and disaster management. Among the crucial aspects of urban areas is the structure of road, which plays a vital role in urban planning, automated navigation, transportation systems and unmanned vehicles [1]. Researchers in the field of RSI processing have a keen interest in extracting road networks, and high-resolution RS data is a valuable resource for real-time road network updates [2]. Thus, presenting a novel road-structure extraction approach from these images aids geospatial information systems (GIS) and intelligent transportation systems (ITS). However, several challenges complicate the extraction of roads from high-resolution RSI [3]. For example, extracting additional features from high-resolution images, such as tree shadows, vehicles on the road, and buildings alongside the road, presents difficulties [4]. Road networks exhibit intricate designs in RSI, with road segments appearing uneven. Accurate road structure extraction from aerial imagery is widely acknowledged as challenging due to the diverse road-type shadows and occlusion resulting from the proximity of trees and buildings. [5]. Previous studies have identified the five key factors for road extraction from aerial images as geometrical factors, including road curvature and length to breadth; radiometric factors [6]; road surface homogeneity and consistent gray color contrast; topological factors, as roads form interconnected networks without abrupt endings for topological reasons; and functional factors, such as the connecting of various regions within a city, including residential and commercial areas [7,8,9,10]. These factors collectively contribute to the road’s overall characteristics, but lighting conditions and obstructions can alter their appearance, adding to the complexities of road extraction [7,8,9,10]. Researchers have turned to artificial intelligence (AI) techniques, utilizing the important usefulness of deep convolutional neural networks (DCNNs) in diverse computer vision (CV) domains, to tackle the extraction of road networks from high-resolution RSI. Convolutional neural networks (CNNs) were first introduced by Yann Le Cun et al. in 1989 as a robust deep learning technique [11]. CNNs have demonstrated exceptional proficiency in the automated extraction of features from various types of data, thus proving their efficacy in computer vision tasks [12,13,14,15]. Simultaneously, progress has been made in quantum technologies.

The discipline of quantum machine learning is rapidly growing and has demonstrated its ability to enhance classical machine learning methods [16,17,18,19,20,21,22,23,24,25], including support vector machines, clustering, and principal component analysis. Quantum convolutional neural networks (QCNNs) are a notable field of study, representing a subset of variational quantum algorithms. QCNNs integrate quantum convolutional layers that employ parameterized quantum circuits to approximate intricate kernel functions within a high-dimensional Hilbert space. Liu et al. (2019) pioneered the development of the first QCNN model for image identification, drawing inspiration from regular CNNs [26]. This groundbreaking work has since sparked further investigation and research in the field, as evidenced by following publications [27,28,29,30,31,32], motivating the application of QCNN with improvement in its basic architecture for road extraction from HRSI.

Significant advancements have been made in extracting high-level features and improving the performance of numerous computer vision tasks, such as object detection, classification, and semantic segmentation [33,34]. These approaches demonstrate superior results compared to traditional methods, particularly when addressing the challenges posed by obstacles and shadow occlusion, geometrical factors, road curvature, length to breadth ratio, and radiometric factors [6] in road extraction from high-resolution imagery.

Therefore, the motivation for converting a CNN model into a quantum-based CNN architecture is significant due to its potential for enhancing performance in many applications, including road extraction and various other cognitive problems. Quantum machine learning models, such as quantum dilated convolutional neural networks (CNNs), leverage quantum circuits to execute convolutions on input data. This approach has the potential to offer improved computational efficiency compared to traditional convolutional layers under specific conditions. Furthermore, quantum machine learning models have the capability to exploit the distinct characteristics of quantum systems in order to execute specific computations more efficiently than classical systems.

## 2. Related Works

Shao et al. [35] presented a novel road extraction network that incorporates an attention mechanism, aiming to address the task of automating the extraction of road networks from large volumes of remote sensing imagery (RSI). Their approach builds upon the U-Net architecture, which leverages spatial and spectral information and incorporates spatial and channel attention mechanisms. In addition, the researchers incorporated a residual dilated convolution module into their approach to capture road network data at various scales. They also integrated residual, densely connected blocks to effectively improve feature reuse and information flow. In a separate study [36], the researcher employed RADANet, an abbreviation for road-augmented deformable attention network, in order to effectively capture extensive interdependencies among particular road pixels. This was motivated by prior knowledge of road morphologies and advancements in deformable convolutions.

Li et al. [37] introduced a cascaded attention-enhanced framework designed to extract roadways with finer boundaries from remote sensing imagery (RSI). The proposed architecture integrates many levels of channel attention to enhance the fusion of multiscale features. Additionally, it incorporates a spatial attention residual block to effectively capture long-distance interactions within the multiscale characteristics. In addition, a lightweight encoder–decoder network is used in order to enhance the accuracy of road boundary extraction. Yan et al. [38] proposed an innovative approach to road surface extraction, incorporating a graph neural network (GNN) that operates on a pre-existing road graph composed of road centerlines. The suggested method exploits the GNN approach for vertex adjustment and employs CNN-based feature extraction to define road surface extraction as a two-sided width inference problem of the road graph. Rajamani et al. [39] aimed to develop an automated road recognition system and a building footprint extraction system using CNN from hyperspectral images. They employed polygon segmentation to detect and extract spectral features from hyperspectral data. CNN with different kernels was used to classify the retrieved spectral features into two categories: building footprints and road detection. The authors introduced a novel deep neural network approach, referred to as dual-decoder-U-Net (DDU-Net), in their study [40]. The authors incorporated global average pooling and cascading dilated convolutions to distill multiscale features. Additionally, a dilated convolution attention module (DCAM) was introduced between the encoder and decoder to expand the receptive field. The authors of reference [41] have proposed a novel road extraction network named DA-RoadNet, which integrates the ability to incorporate semantic reasoning. The primary architecture of DA-RoadNet consists of a shallow network that connects the encoder to the decoder. This network incorporates densely connected blocks in order to address the issue of road infrastructure data loss resulting from several down-sampling procedures. Hou et al. [42] proposed a route extraction approach for RSI using a complementary U-Net (C-UNet) with four modules. They introduced an MD-UNet (multi-scale dense dilated convolutional U-Net) to identify complementary road regions in the removed masks, after the standard U-Net was employed for rough road data extraction from RSI and generated the initial segmentation result. Table 1 and Table 2 summarize various proposed architectures from the last three years and offer a comparison based on various key parameters, respectively.

The practical execution of many quantum circuits still poses challenges. QCNNs face computational difficulties due to the need to execute additional circuits for quantum operations and gradient calculations [28,29]. The utilization of quantum filters that possess trainable characteristics further exacerbates this concern. Unlike classical CNNs, QCNNs often lack vectorization capabilities on the majority of quantum devices, hence impeding their scalability [43,44].

To reduce the runtime complexity of QCNN, two main approaches are prominent. Firstly, dimension reduction techniques such as principal component analysis (PCA) and autoencoding can reduce the required qubits, but they may constrain the model’s expressiveness [45,46]. Secondly, the efficient conversion of classical data into quantum states is pursued through encoding methods. Amplitude encoding conserves qubits but relies on complex quantum circuits [47,48]. Conversely, angle encoding and its variants maintain consistent circuit depth but may be less efficient for high-dimensional data [32,49,50]. A hybrid encoding approach strikes a balance between qubit usage and circuit depth [46], while threshold-based encoding simplifies quantum convolution but may have limitations on real quantum devices [28].

Considering the various challenges that have been thoroughly examined and the subsequent advancements made, this study presents an unconventional quantum-classical architecture called the quantum dilated convolutional neural network (QDCNN) for road extraction with the Archimedes tuning process (ATP) from high-resolution remote sensing images. Initially, our proposed methodology benefited from previous architectures [26,28], and for the dilated convolutional neural network, it uses the architecture described in [51] and introduces a new strategy to decrease the computing expenses of QCNN in the use of a quanvolutional layer [28], drawing inspiration from dilated convolution techniques in deep learning. The utilization of dilated convolution, which was initially devised for discrete wavelet transformations [52], has become increasingly prominent in various fields, such as semantic segmentation [21,53,54,55,56,57], object localization [58], sound classification [59] and time-series forecasting [60,61]. The utilization of dilated convolution in QDCNNs effectively increases the filter context, resulting in improved computing efficiency without any additional parameters or complexity.

The literature survey uncovered three research gaps:

- The limited investigation of quantum-inspired methodologies for the extraction of road features from remote sensing images.
- The proposed ATP-QDCNNRE method described in Section 3.1 attempts to address the integration of quantum-inspired dilated convolutions, exploring quantum computing concepts and optimizing dilated convolutions for long-range dependencies for road extraction from road datasets.
- Failures in the efficient use of dilated convolutions and automated hyperparameter modifications to achieve good road semantic segmentation using deep learning models. Employing automated hyperparameter tuning in Section 3.2, and developing a fully automated road extraction system.

In summary, the present study makes two significant contributions:

- The proposed ATP-QDCNNRE method attempts to address the integration of quantum-inspired dilated convolutions, explore quantum computing concepts, optimize dilated convolutions for long-range dependencies, employ automated hyperparameter tuning, and develop a fully automated road extraction system.

Implementing quanvolutional layers in the dilated convolutional neural network is a novelty of the proposed model. Various parameters of quantum circuits, such as qubits and gates, are highly tunable parameters that need to be taken into account to interpret results.

- The study performs experiments utilizing the Massachusetts road dataset to showcase the enhanced performance of the QDCNN model.

**Table 1:** Comparative study of literature review on road extraction in the last three years.

Authors [Citations]	Year	Methodology	Challenges
Tao, J et al. [62]	2023	SegNet; Road extraction based on transformer and CNN with connectivity structures.	Narrowness, complex shape, and broad span of roads in the RS images; the results are often unsatisfactory.
Yin, A et al. [63]	2023	HRU-Net: High-resolution remote sensing image road extraction based on multi-scale fusion	Shadow, occlusion, and spectral confusion hinder the accuracy and consistency of road extraction in satellite images.
Shao, S et al. [35]	2022	Road extraction based on channel attention mechanism and spatial attention mechanism were introduced to enhance the use of spectral information and spatial information based on the U-Net framework	To solve the problem of automatic extraction of road networks from a large number of remote sensing images.
Jie, Y et al. [64]	2022	MECA-Net is a novel approach for road extraction from remote sensing images. It incorporates a multi-scale feature encoding mechanism and a long-range context-aware network.	The scale disparity of roads in remote sensing imagery exhibits significant variation, with the identification of narrow roadways posing a challenging task. Furthermore, it is worth noting that the road depicted in the image frequently encounters obstruction caused by the shadows cast by surrounding trees and buildings. This, in turn, leads to the extraction results being fragmented and incomplete.
Li, J et al. [65]	2021	Proposed an innovative cascaded attention DenseUNet (CADUNet) semantic segmentation model by embedding two attention modules, such as global attention and core attention modules	To preserve the integrity of smoothness of the sideline and maintain the connectedness of the road network; also to identify and account for any occlusion caused by roadside tree canopies or high-rise buildings.
Wu, Q et al. [66]	2020	Based on densely connected spatial feature-enhanced pyramid method	Loss of multiscale spatial feature.

**Table 2:** Presents comparative results of the methods shown in Table 1.

Authors	Results Based on Various Parameters Used by Authors					
	Overall Accuracy (%)	Accuracy (%)	Precision (%)	Recall (%)	F1-Score (%)	IoU (%)
Tao, J et al. [62]	--	--	87.34	92.86	90.02	68.38
Yin, A et al. [63]	--	--	80.09	84.85	82.40	78.62
Shao, S et al. [35]	--	98.90	78.40	77.00	76.40	63.10
Jie, Y et al. [64]	--	--	78.39	79.41	89.90	65.15
Li, J et al. [65]	98.00	--	79.45	76.55	77.89	64.12
Wu, Q et al. [66]	--	--	90.09	88.11	89.09	80.39

## 3. The Proposed Methodology

**Figure 1:**
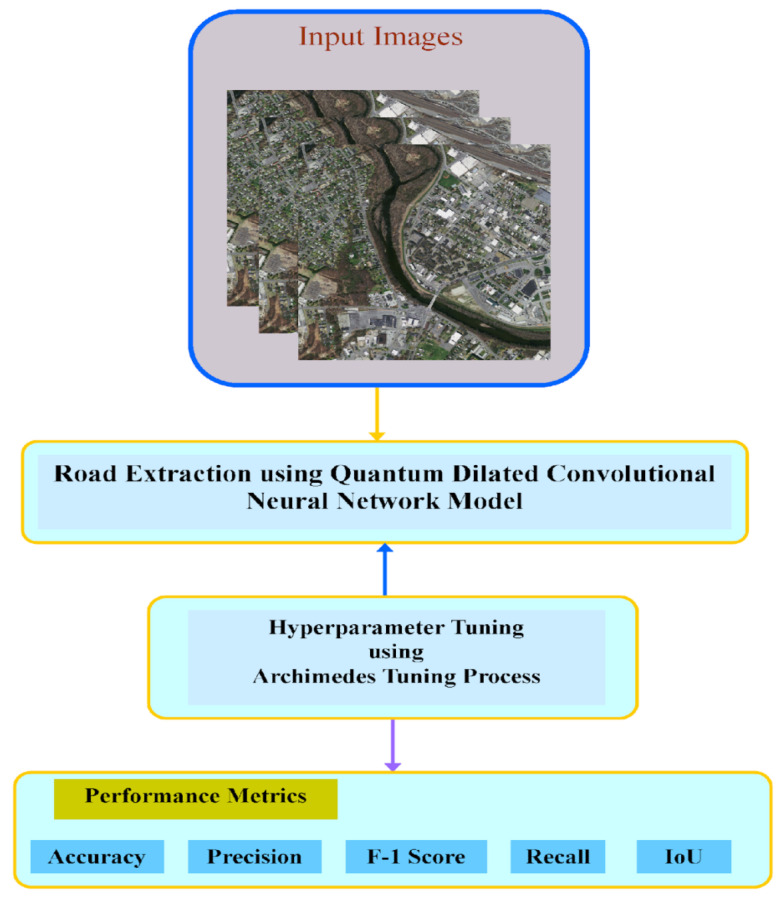
Workflow of ATP-QDCNNRE algorithm.

**Figure 2:**
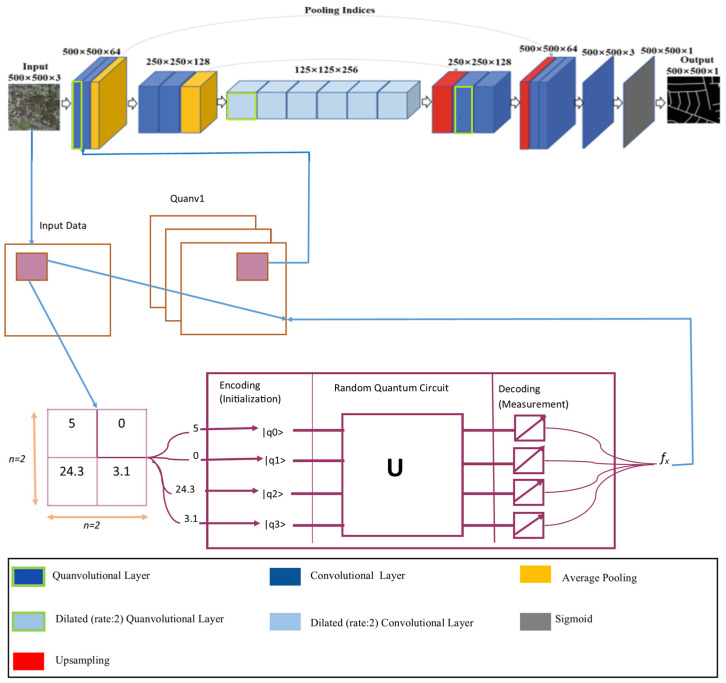
Detailed architecture of QDCNN model.

In order to turn a classical deep learning semantic segmentation architecture into a quantum-enabled convolutional neural network (CNN) overview of the workflow is shown in Figure 1 classical convolutional layers are replaced with quantum convolutional layers, as shown in Figure 2. Quantum convolutional layers consist of a collection of N quantum filters that function similarly to classical convolutional layers. These filters generate feature maps by locally modifying the input data. Various optimization approaches, such as the various quantum eigen solver (VQE) or the quantum approximate optimization algorithm (QAOA), including the integration of Archimedes optimization algorithms (AOA), are used to optimize the parameters of quantum filters.

In real-world situations, the conversion procedure may encompass multiple stages, including defining the quantum circuit for the quantum convolutional layer, initializing circuit parameters, and optimizing those parameters using an appropriate optimization algorithm. The complexity of this procedure may vary based on several aspects, including the specific architecture employed, the quantity and quality of the training data, and the specific demands of the given task.

This paper presents the unique ATP-QDCNNRE system utilizing the Archimedes optimization algorithm (AOA) [67], designed for effective and automated road extraction from RSI. The primary objective of the ATP-QDCNNRE system is to generate high-resolution road segmentation maps through the application of deep learning (DL) techniques and a hyperparameter tuning methodology with the integration of a quanvolutional layer into a dilated convolutional neural network proposed by [28,51]. The proposed technique consists of two main processes: road extraction using QDCNN with hyperparameter adjustment using ATP and implementation of a quanvolutional layer into a dilated neural network with the help of a quantum tool in Python called PennyLane [68]. The utilization of the quantized dilated convolutional neural network (QDCNN) architecture in the ATP-QDCNNRE system is primarily aimed at road extraction, as it is developed to produce improved road segmentation. The performance of road extraction can be improved by the QDCNN through its efficient acquisition of local and global road data, achieved by employing dilated convolutions. Furthermore, the ATP-QDCNNRE system integrates an ATP-based methodology for tuning hyperparameters, aiming at improving the hyperparameters of the deep learning model. Through the adaptive operator adjusting technique of the ATP method, the hyperparameters are dynamically tuned during the training phase. This adaptive approach tailors the deep learning (DL) model specifically for road extraction, leading to enhanced segmentation results. Figure 1 provides an overview of the ATP-QDCNNRE system, offering a visual representation. The figure showcases the integration of the sequential stages of the ATP-QDCNNRE system, including the processes of QDCNN-based road extraction and ATP-based hyperparameter tuning.

### 3.1. Road Extraction Using QDCNN Model

In the automated road extraction process, the QCNN approach was employed in this work. The QDCNN model proposed in this study features a three-tier-based architecture, integrating a quantum layer, a convolution layer, and a dilated layer for road extraction processes [69]. It utilizes a bi-directional cross-entropy function as the loss parameter for its classification layer, enabling effective learning and performance evaluation. Figure 2 illustrates the infrastructure of the QDCNN model.

Firstly, the baseline model as described by [51] utilizes the DCN algorithm, which performs four standard convolution operations and two average pooling operations on the input image. Consequently, the dimensions of the feature map are reduced to one-fourth of the dimensions of the original image. Subsequently, the system executes six dilated convolution operations on the resulting feature maps with a dilation rate of 2. Afterward, the feature maps are transmitted to the decoding stage, where two up-sampling operations restore them to their original dimensions before the initial image. Finally, the resulting output is assigned a corresponding probability value for pixel classification, achieved by employing a convolutional layer with a single channel and convolution kernel size of 1 × 1.

In the proposed model, the first classical convolutional layer is transformed into a quanvolutional layer using the approach described in [28]. The rest of the network remains the same. Initially, implementation required a quantum computing device; however, since we do not have access to one, various quantum computing simulators were applied for implementation. Ultimately, PennyLane [68], a Python plugin, was utilized to implement the concept and produce the desired results.

#### 3.1.1. Convolutional Layer

The term “convolutional operation” denotes a linear function that performs the aggregation of weights linked to the input, playing a crucial role in the functioning of Convolutional Neural Networks (CNNs). The prior source image was represented by *i* and the resultant mapping feature was denoted by j. The mapping feature was produced by the filter (*f*), and the implemented technique was applied to the source images *i*.

(1) $$[a,b]=\sum_{p}\sum_{k}f\left[p,k\right].i[a+p,b+k],$$

In Equation (1), a and b represent the indices that are part of J. Compared to the source image, the resultant mapping feature typically has a lower spatial resolution, depending on the convolution approach used. To apply the filter, the borders of the source input were enclosed with pixels having a zero value, a process known as padding. Generally, the spatial resolutions ${Out}_{g}$ and ${Out}_{i}$ are the most recent mapping features, and the g×h kernel extracted from the ${in}_{g}\times {in}_{i}$ source image was computed as follows:

(2) $${Out}_{g}=\left(\frac{{in}_{g}-g+2p}{s}\right)+1,$$

(3) $${Out}_{i}=\left(\frac{{in}_{i}-h+2p}{s}\right)+1,$$

where as p and s refer to the padding stride correspondingly.

#### 3.1.2. Dilatable Convolution

According to the findings presented in reference [56], it is observed that the use of standard pooling and convolution processes results in a reduction in the resolution of feature maps. The issue is resolved by introducing a dilation rate parameter in dilated convolution. In contrast to conventional convolution, this method preserves the kernel size while enlarging the receptive field. This feature is highly advantageous for activities that require the preservation of spatial detail.

**Figure 3:**
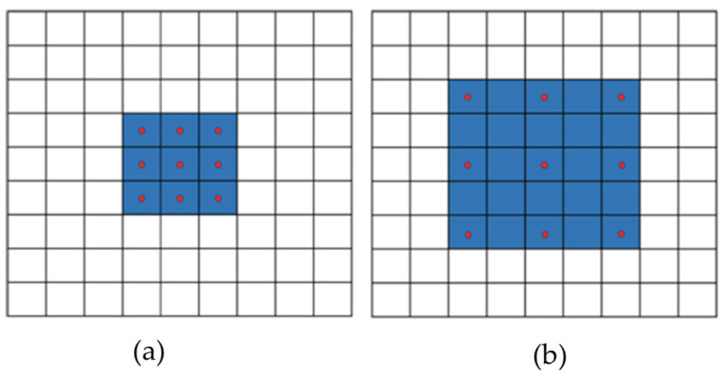
Illustrates two types of convolution. (**a**) The first type of convolution is referred to as ordinary convolution, involving a 1-dilated convolution. (**b**) The second type of convolution is known as dilated convolution, specifically a 2-dilated convolution.

Figure 3 depicts a standard convolution alongside a dilated convolution, where the latter has a dilation rate of 2. The utilization of dilated convolution is observed to enhance the receptive field of the convolution kernel while maintaining a constant number of parameters. Simultaneously, it has the capability to maintain the dimensions of the feature maps unchanged. The receptive field of a convolution kernel with dimensions 3 × 3 and a dilation rate of 2 is equivalent to that of a convolution kernel with dimensions 5 × 5. However, the former requires only 9 parameters, which accounts for just 36% of the parameter count of the latter.

The dilated convolutional layer is a specific sort of convolutional layer that incorporates gaps between consecutive kernels, resulting in an expanded kernel. Dilation rate controls input pixel sampling frequency in this layer, which is shown in Figure 3.

(4) $$\left[a,b\right]=\sum_{p}\sum_{k}f\left[p,k\right].i\left[a+p.d,b+k.d\right],$$

Without adding learnable parameters, dilated convolution records a broader receptive field than classical convolution using a similar kernel.

(5) $${Out}_{g}=\left(\frac{{in}_{g}-g-(g-1)(d-1)+2p}{s}\right)+1,$$

(6) $${Out}_{i}=\left(\frac{{in}_{i}-h-(h-1)(d-1)+2p}{s}\right)+1,$$

The above mentioned formula demonstrates that, for the given set of hyperparameters, dilated convolution usually produces a small mapping feature compared to typical convolution.

#### 3.1.3. Fundamentally Different Quantum Convolution by Quanvolutional Filter

Quantum growing concepts distinguish quantum convolution (QC) from common convolution. In quantum convolution, there are three modules: encoder, entanglement, and decoder.

- Encoder model: At present, the data are encoded to a quantum state, and the encoded data are examined using QC circuits. One of the variable encoding methods that can be exploited to encode information is the Hadamard gate (H). The encoder function represented by E(a) transforms an initial state into a uniform superposition state *i*, specifically focusing on the data vector.
  (7) $$⎹\;i\;⦒=E\left(a\right)⎹\;0\;⦒,$$

- Entanglement state: The encoder quantum state generated in the preceding model element has an effect on the single- and multi-qubit gates inside this module. Commonly utilized multi-qubit gates in quantum computing encompass CNOT gates and parametrically controlled rotation. The utilization of both single-qubit and multi-qubit gates in a composite manner results in the formation of parameterized layers. These layers can be further optimized to acquire assignment properties. If the unitary operations of the entanglement modules are all denoted by (θ), then the resulting quantum state can be represented as follows:
  (8) $$⎹⎹\;i,\theta \;⦒=U\left(\theta \right)⎹\;i\;⦒,$$

- Decoder model: Subsequently, local variables, such as the Pauli Z operator, are estimated for the previous modules. The predictable value θ of local variables is attained by the following equation:
  (9) $$⦑\;i,\theta \;⦒=A^{ⴲ\;x}⎹\;i,\theta \;⦒,$$

To create a quantum state-to-vector mapping, $f\left(i,\theta \right)$

(10) $$⎹\;i,\;\theta \;⦒=f\left(i,\theta \right),$$

In Equation (10), f(i,θ) represent the input for QCNN. During the presented method, quantum and classical layers are integrated, and the quantum circuit analysis are exploited throughout. The primary distinction between common networks and the presented quantum convolutional neural networks (QCNNs) is the incorporation of dilated convolutional layers, resulting in the development of the quantum dilated convolutional neural network (QDCNN). The QDCNN possesses two distinct advantages. The QDC layer exhibits a reduced number of iterations for the quantum kernel’s traversal over the image, attributed to its expansive receptive fields. Additionally, the QDC layer employs usual methods to decrease the spatial resolution of generated mapping features owing to its enhanced receptive field, as shown in Figure 2.

#### 3.1.4. Network Design of (Quantum Circuit) Quanvolutional Layer

The design of quanvolutional networks using quantum neural networks (QNNs) represents an extension of CNN, incorporating an extra transformational layer known as the quanvolutional layer. The integration of quanvolutional layers should be identical to that of classical convolutional layers, hence enabling users to:

- assign the variable p to represent an arbitrary integer value, denoting the number of quanvolutional filters in a specific quanvolutional layer;
- add multiple additional quanvolutional layers on top of any existing layer within the network architecture.

The layer-specific configurational attributes encompass several aspects, such as encoding and decoding methods, as well as the average number of quantum gates per qubit in the quantum circuit.

By meeting these criteria, the quanvolutional filter is expected to possess a high degree of generalizability and can be implemented with equal ease in any architecture, similarly to its classical predecessor. The determination of the quantity of layers, the sequence of their implementation, and the exact parameters associated with each layer are fully dependent on the specifications provided by the end user. The visual representation of the universality of QNNs is depicted in Figure 2. A new feasible model can be built by modifying, eliminating, or incorporating layers according to preference. The network design shown in Figure 2 would retain its structure if the quanvolutional layer were substituted with a convolutional layer consisting of 32 filters. Similarly, the convolutional layer could be replaced with a quanvolutional layer, including 128 quanvolutional filters, while maintaining the general structure of the network. The distinction between the quanvolutional and convolutional layers is contingent upon the manner in which the quanvolutional filters perform calculations.

### 3.2. Hyperparameter Tuning Using ATP

In this work, the ATP- hyperparameter tuning process based on the Archimedes optimization algorithm (AOA) [70] was utilized. ATP is a robust and novel optimization approach embodying the principles of Archimedes [70]. The outcomes of the experiment showed that ATP can effectively address optimizer issues and solve near-optimum or fetch optimum problems in a shorter period. The ATP mathematical formula consists of several stages, as given below:

*Stage1*: Initialization. A group of random individuals was generated and kept in the location, as follows:

(11) $$⎹O_{i=}={lb}_{i}+rand\times \left({ub}_{i}-{lb}_{i}\right),\;i=1,2,3\ldots \ldots .N$$

In Equation (11), $O_{i}$ represents the $i^{th}$ agent’s (object’s) location of total agents N, $ub_{i}$ and $\;lb_{i}$ denotes the upper and lower boundaries of $i^{th}$ agents correspondingly, and rand specifies the Dimn dimension random vector in the interval.

(12) $${dens}_{i}=randm,$$

(13) $${Vol}_{i}=randm,$$

where $vol_{i}$ represents the volume of agent, and $i^{th}$ and ${dens}_{i}$ denotes the density.

(14) $${accn}_{i}={ib}_{i}+randm\times ({ub}_{i}-{lb}_{i}),$$

In Equation (14), $ac{cn}_{i}$ denotes the acceleration, $ub_{i}$ and $lb_{i}$ indicate the upper boundaries of $i^{th}$ agents correspondingly, and randm shows the Dimn -dimension random value in the interval.

*Stage2*: Updating Density and Volume. Both density and volume are used as in Equations (15) and (16)

(15) $${dens}_{i}^{t+1}={dens}_{i}^{t}+randm\times ({dens}_{best}-{dens}_{i}^{t}),$$

(16) $${Vol}_{i}^{t+1}={Vol}_{i}^{t}+randm\times ({Vol}_{best}-{Vol}_{i}^{t}),$$

where $vol_{best}$ and $de{ns}_{best}$ indicate the optimum volume and density obtained at point t, and $vol_{i}^{t}$ and $de{ns}_{i}^{t}$ use the terms “volume” and “density” of $j^{th}$ operatives in position t.

*Stage3*: Transfer Operators and Density Factors. Here, collisions occur between the individuals and the agent, and then the agent tries to reach a state of equilibrium. A transfer operator TF is used to shift between exploration and exploitation:

(17) $$TF=exp\left(\frac{t-t_{max}}{t_{max}}\right),$$

In Equation (17), t and $t_{max}$ denote the existing and maximal iteration count. Furthermore, a reduction in density factors was added to support ATP in fetching a near-optimal solution, as follows:

(18) $$d^{t+1}=exp\left(\frac{t_{max}-t}{t_{max}}\right)-\left(\frac{t}{t_{max}}\right),$$

*Stage 4*: Exploration of where the individuals’ collision occurs. If the TF is less than 0.5, then arbitrary material is selected and the acceleration of the agent i′s is enhanced, as follows:

(19) $${accn}_{i}^{t+1}=\frac{{dens}_{mr}+{vol}_{mr}\times {accn}_{mr}}{{dens}_{i}\times {vol}_{i}},$$

In Equation (19), ${vol}_{mr},\;ac{cn}_{mr}$, and $de{ns}_{mr}$ indicate the volume, acceleration, and density of an element that has been randomly generated, and $vol_{i},ac{cn}_{i}$, and $de{ns}_{i}$ encapsulate the volumetric, acceleration, and density of $i^{th}$ agents.

*Stage 5*: Exploitation where no individual collision takes place. When the TF is greater than approximately 0.5, the acceleration of agent i′s is upgraded using the following equation:

(20) $${accn}^{t+1}=\frac{{dens}_{best}+{vol}_{best}\times {accn}_{best}}{{dens}_{i}\times {vol}_{i}^{t+1}},$$

In Equation (20), $ac{cn}_{best},\;de{ns}_{best},\;\mathrm{and}\;vol_{best}$ show the specific rates of acceleration, density, and volume.

*Stage 6*: Normalizing acceleration: The process of normalizing acceleration involves the utilization of Equation (21):

(21) $${accn}_{i-norm}^{t+1}=u\times \frac{{accn}_{i}^{t+1}+\min(accn)}{\max\left(accn\right)-\min(accn)}+1,$$

where max(accn), and min(accn) denote the maximal and minimal acceleration correspondingly. $ac{cn}_{i-norm}^{t+1}$ represents the percentage individuals who are shifting steps, and l and u indicate lower as well as upper bounds of normalization that equal 0.1 and 0.9 correspondingly.

*Stage 7*: Location updating: Equation (20) updates an individual’s location if TF is less than 0.5; otherwise, Equation (23) is utilized.

(22) $$g_{i}^{t+1}=g_{i}^{t}+c_{1}\times randm\times {accn}_{i-norm}^{t+1}\times d\times (g_{randm}-g_{i}^{t}),$$

(23) $$g_{best}^{t+1}=g_{best}^{t}+F\times c_{2}\times randm\times {accn}_{i-norm}^{t+1}\times d\times (T\times x_{best}-g_{i}^{t}),$$

Here, $g_{i}^{t}$ signifies the $i^{th}$ agents (object) at iteration, $t,g_{best}^{t}$ denotes the optimum agent at t iteration, and d indicates the dimensionality; $c_{1}$ and $c_{2}$ are constants. T signifies time function and equal $c_{3}\times TF$, where $c_{3}$ falls within [$c_{3}\times 0.3,1$] and takes a fixed ratio from the optimal place. Subsequently, it diminishes to the spatial separation between the present and ideal locations, thereby denoting a directional movement using flag *F* and *p*. The Probability is calculated using the equation:

(24) $$F=\left\{\begin{array}{c} +1,\;if\;p<0.5\\ -1,\;if\;p>0.5 \end{array}\right.,$$

where $P=2\times randm-C_{4}$. Each agent is evaluated using objective function f, and the optimal values are remembered. $x_{best,}{\;dens}_{best,\;}{vol}_{best,}$ and ${accn}_{best}$, are assigned. Pseudo code for the complete ATP based on AOA is provided as follows for better significance.

## 4. Data and Results

### 4.1. Data Collection

The evaluation of the road extraction results obtained from the ATP-QDCNNRE method is conducted on the Massachusetts road [71] dataset.

#### The Massachusetts Road Dataset and Its Preprocessing

**Figure 4:**
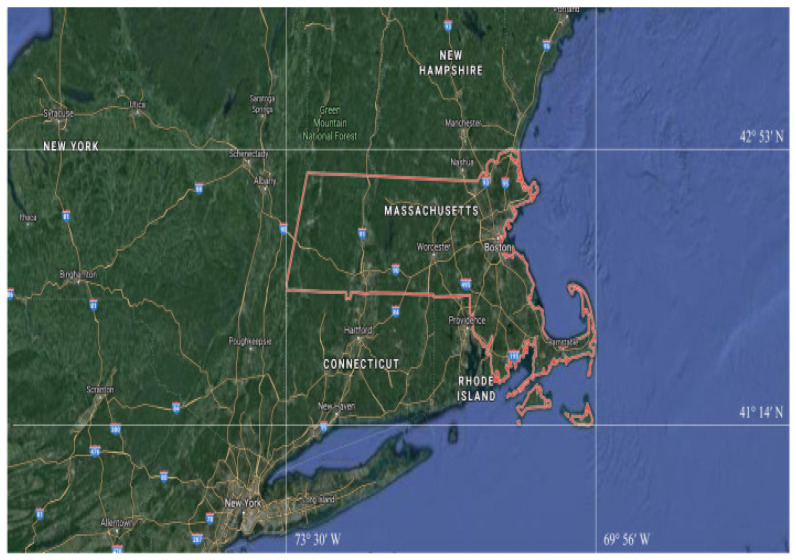
Location of Massachusetts (U.S) is outlined in the map.

**Figure 5:**
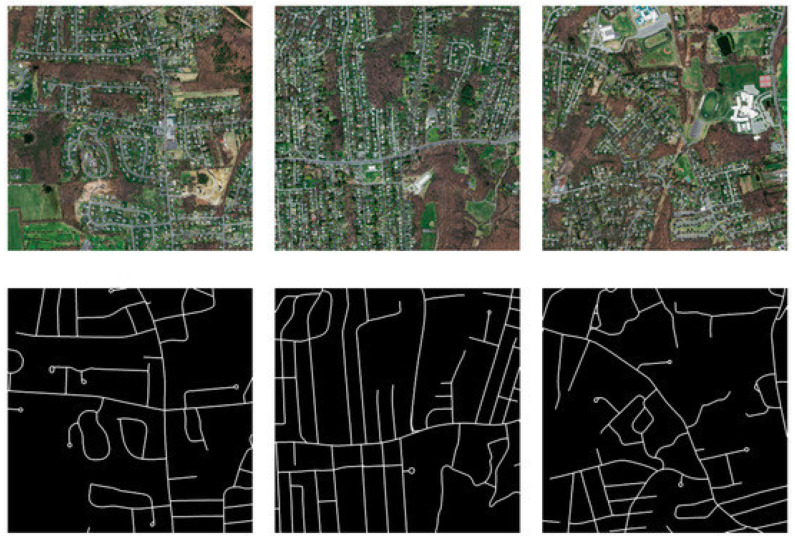
Sample images with corresponding labels of Massachusetts road dataset.

The Massachusetts road dataset [71] is globally recognized as the most extensive publicly accessible road dataset. The dataset covers a wide variety of urban, suburban, and rural regions and covers an area of over 2600 square kilometers, as shown in Figure 4. The dataset comprises 1171 images, which are further categorized into subsets, including 1108 training images, 14 validation images, and 49 test images. Additionally, corresponding label images are provided, as depicted in Figure 5. Each image has dimensions of 1500 × 1500 pixels, with a resolution of 1.2 m per pixel. The dataset comprises a range of distinct attributes, including road networks, grassland areas, forested regions, and built structures.

**Figure 6:**
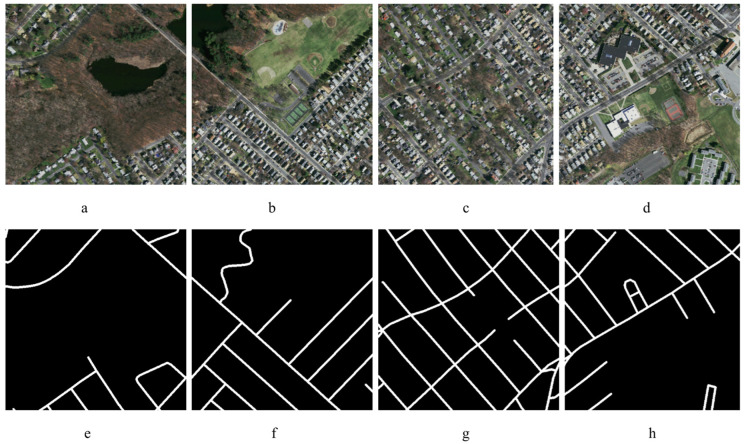
Sample cropped images with corresponding labels of Massachusetts road dataset (**a**–**d**) and (**e**–**h**) respectively.

**Figure 7:**
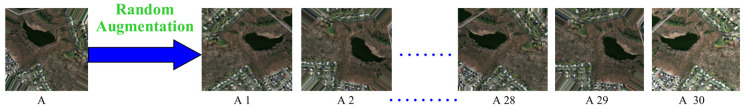
Sample augmented images with corresponding labels of Massachusetts road dataset. From each patch like A, generated 30 images from A1–A30, after applying random augmentation.

The dataset preprocessing follows the approach outlined in [8], with some modifications in the cropping size, as illustrated in Figure 6, and in the augmentation process. In the previous study [8], 100 images were generated from a single image, but in this study, only 30 images were generated, as shown in Figure 7, to augment the sample size. The remote sensing images from the training and validation sets, along with their related label images, were divided into image samples of size 500 × 500 pixels. Based on the principles governing the partitioning of datasets, all divided sample data were randomly allocated into two distinct subsets: a training set and a validation set, with a ratio of 4:1. During the process of sample examination, it was necessary to remove a certain number of interfering images along with their matching label images. The training set had a total of 14,086 images, each with dimensions of 500 × 500 pixels. Similarly, the validation set consisted of 4695 images, all with dimensions of 500 × 500 pixels.

### 4.2. Experimental Results

All experiments adopted the same parameter initialization method and optimizer during the training process, with differences in the parameters for QDCNNRE, as shown in Table 3. In the experiment, raw image data were transformed into feature maps using a non-trainable quantum filter. Each model was trained for 50 epochs using a mini-batch size of 32 and the Adam optimizer with a learning rate of 0.01. The batch size was reduced to four in order to mitigate the computational expenses associated with training parametric quantum circuits in the trainable quantum filter. PennyLane [68], Qulacs [72], and PyTorch [73] were utilized in this study. PennyLane is a Python-based system that is open source and facilitates automatic differentiation for hybrid quantum-classical computations. The software is compatible with widely used machine learning frameworks, such as TensorFlow and PyTorch. Additionally, it offers a comprehensive collection of plugins providing access to various quantum devices, including simulators and hardware, from multiple suppliers, such as IBM, Google, Microsoft, Rigetti, and QunaSys. The PennyLane-Qulacs plugin is referenced as [74]. Due to the substantial number of quantum circuit executions required in the parameter-shift rule scheme, we chose to train all hybrid models using the built-in PennyLane simulator default.qubit. This simulator supports the backpropagation approach for the PyTorch interface. The experiments were conducted on a Windows operating system with Intel (R) Xeon (R) CPU E5-2687 V4 @ 3.00 GHz as the CPU. The GPU model used was NVIDIA GRID RTX8000-12Q with 12 GB of memory.

The parameters of the quantum circuit were optimized using various methods, such as the variation quantum Eigen solver (VQE), the quantum approximate optimization algorithm (QAOA), and the Archimedes optimization algorithm. Hyperparameters for a regular CNN in semantic segmentation include the size of kernels, the number of kernels, the length of strides, and the pooling size, all of which directly affect the performance and training speed of CNNs. Hyperparameters for a quantum dilated CNN for semantic segmentation include learning rate, mini-batch size, momentum, optimizer, and weight decay. Some of these hyperparameters are provided in Table 3 with their descriptions.

#### 4.2.1. Evaluation Method

The cross-entropy loss function is commonly employed in deep convolutional neural networks for solving two-class problems. It quantifies the likelihood of belonging to a particular class [20]. Therefore, the loss function employed in our study was based on [20].

(25) $$L=-\frac{1}{n}\sum_{i=1}^{n}y_{i}loga_{i}+\left(1-y_{i}\right)\log\left(1-a_{i}\right),$$

where $y_{i}$ indicates the real category of the input $x_{i}$, in which $y_{i}^{1/4}$ means that the ith pixel belongs to the road. The $a_{i}$ is the category of the pixel in output image, which is modeled by the following sigmoid function:

(26) $$a_{i}=\frac{1}{1+e^{{-z}_{i}}},$$

where $z_{i}$ denotes the input of the last layer.

The accuracy of road extraction outputs achieved by each network was assessed using four assessment metrics: intersection over union (IoU), precision, recall, and F1. The IoU metric is a measure of the proportion between the intersection and union of prediction outcomes and labels. Precision is a metric that quantifies the ratio of accurately predicted pixels to the total number of pixels anticipated as roads. The term “recall” refers to the ratio of accurately anticipated road pixels to the total number of road pixels. The F1 score is a comprehensive evaluation statistic that represents the harmonic mean of precision and recall. The calculation algorithms for the four aforementioned evaluation measures are presented below:

(27) $$IoU=\frac{TP}{TP+FP+FN},$$

(28) $$Precision=\frac{TP}{TP+FP},$$

(29) $$Recall=\frac{TP}{TP+FN},$$

(30) $$F1=\frac{2\times Precision\times Recall}{Precision+Recall},$$

In the present study, the term “precision” is defined as the proportion of accurately predicted road pixels to the total number of pixels anticipated as roads, as represented by Equation (28). The term “recall” pertains to the proportion of pixels that are accurately classified as roads, relative to the total number of pixels that are actually labeled as roads, as represented by Equation (29). The F1 score is a robust evaluation metric that combines the precision and recall measures, as depicted in Equation (30). In the context of prediction evaluation, true positive (TP) denotes the accurate identification of positive cases, false positive (FP) represents the erroneous identification of negative cases as positive, and false negative (FN) signifies the inaccurate identification of positive cases as negative.

The measurement index of classical (MIoU) is a well-established metric for evaluating the performance of semantic segmentation algorithms. Its calculation is defined by Equation (10), where *K* denotes the total number of categories. In this particular study, K was set to 1, with K+1 representing the number of categories encompassing both foreground and background. The variable *pij* represents the count of objects predicted as belonging to category *j*, when they actually belong to category i. Moreover, IoU (intersection over union) serves as the sole evaluation metric for road segmentation.

(31) $$MIoU=\frac{1}{K+1}\sum_{i=0}^{k}\frac{P_{ij}}{\sum_{j=0}^{K}p_{ij}+\sum_{j=0}^{K}P_{ij}-P_{ii}},$$

#### 4.2.2. Experimental Result Analysis

**Figure 8:**
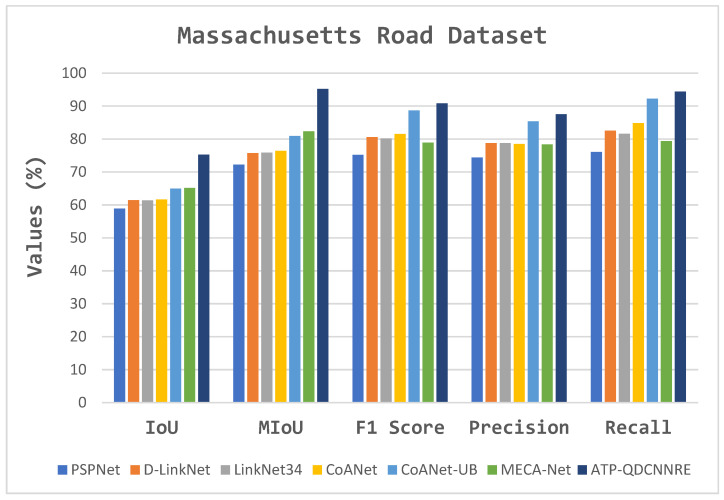
Graphical representation of comparative results described in Table 4.

**Figure 9:**
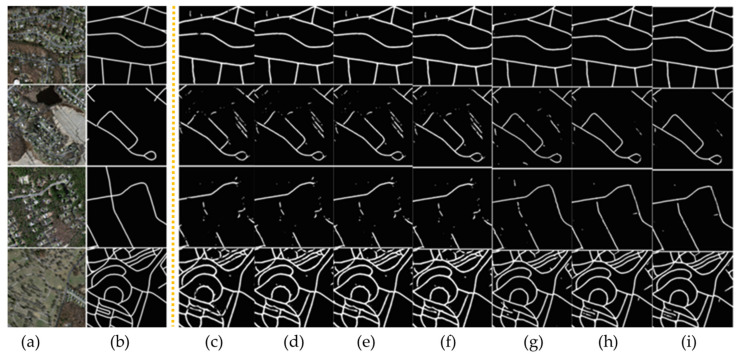
Segmentation results of ATP-QDCNNRE on the Massachusetts road dataset. (**a**) The original images; (**b**) their corresponding ground truth. Visual results of various state-of-the-art models are shown as: (**c**) PSPNet (**d**) D-LinkNet (**e**) LinkNet 34 (**f**) CoANet (**g**) CoANet-UB (**h**) MECA-Net and (**i**) ATP-QDCNNRE (Ours).

In the context of comparative experiments, the proposed method was evaluated against various extraction approaches. The comparative analysis of several methodologies for the road dataset is presented in Table 4, Figure 8 and Figure 9.

In Table 4, the techniques categorized as classical approaches do not employ the framework of convolutional networks. Among deep-learning-based methods, the metrics demonstrate that encoder–decoder architectures, particularly U-Net-like networks, exhibit favorable performance for semantic segmentation. This substantiates the suitability of U-Net-like structures in comparison to existing literature works. The selected model, QDCNNRE, bears resemblance to the U-Net architecture, but incorporates a quanvolutional layer and a dilated convolutional layer as additional components. The addition of a quanvolutional layer, as shown in Figure 2, is instrumental. Table 4 demonstrates that the proposed ATP-QDCNNRE technique exhibited superior performance among all the techniques.

The visual findings provided in Figure 9c–i have been utilized to analyze and evaluate the segmentation performance on the Massachusetts road dataset with many state-of-the-art models. The initial images, denoted as Figure 9a, along with their corresponding ground truth representations in Figure 9b, serve as the foundation for visually assessing the segmentation outcomes. Among the range of advanced models, such as PSPNet, D-LinkNet, LinkNet 34, CoANet, CoANet-UB, and MECA-Net, the ATP-QDCNNRE (Figure 9i) stands out by demonstrating significant improvements in the segmentation task. The visual outcomes indicate that ATP-QDCNNRE demonstrates better performance in capturing road structures and their intricate characteristics, leading to a more effective segmentation outcome in comparison to other similar methods. Table 4 presents a quantitative analysis of the segmentation results, which is expected to corroborate the visual findings. Specifically, the metrics presented in Table 4 indicate effective outcomes for the ATP-QDCNNRE approach. The cumulative data suggest that ATP-QDCNNRE has superior performance compared to various state-of-the-art models in the area of road segmentation.

**Figure 10:**
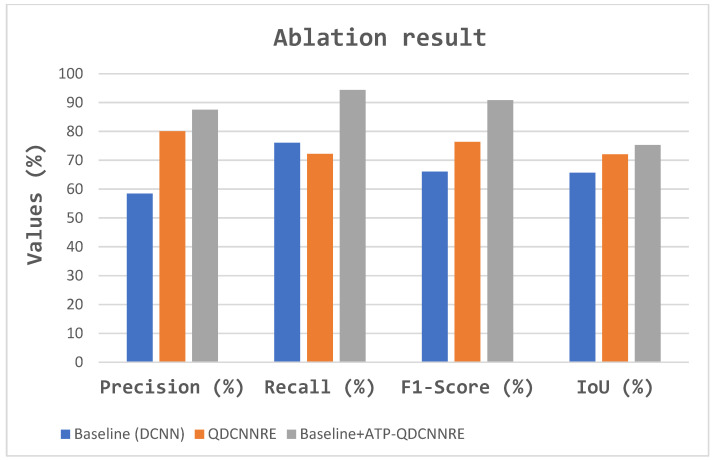
Visualization of ablation experimental process.

**Figure 11:**
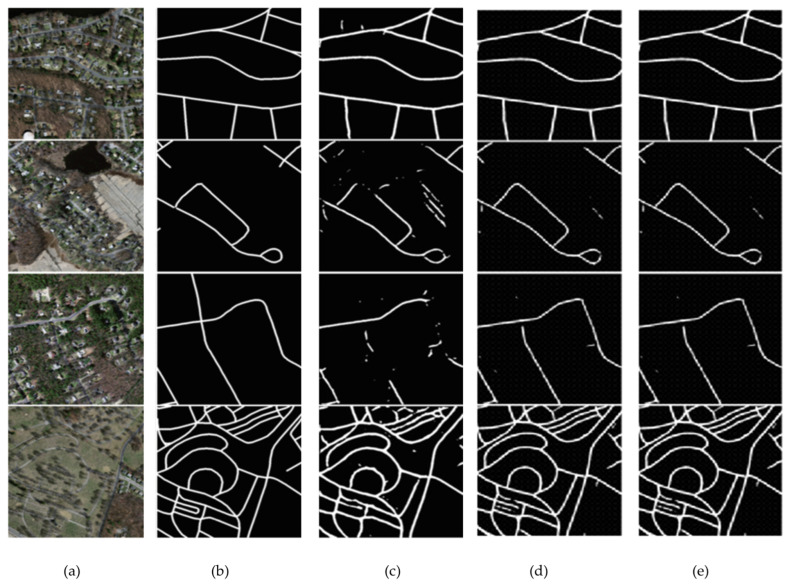
Visual result of the ablation study: (**a**) original image, (**b**) ground truth, (**c**) Baseline DCNN, (**d**) DCNN+ quanvolutional layer, and (**e**) ATP-QDCNNRE.

A study involving an ablation study was conducted to evaluate the functionality of the modules. Table 5 presents a quantitative comparison of the ablation study conducted on the Massachusetts dataset, visually shown in Figure 10 and Figure 11. The Baseline network refers to the modified dilated convolutional network (DCNN) architecture depicted in Figure 2. The Baseline-DCNN incorporates quantum to become the QDCNN module at the top of the QDCNNRE. The baseline-QDCNNRE method employs the Archimedes tuning process (ATP) coupled with the Archimedes optimization algorithm (AoA) to automatically tune the quantum circuit parameters, such as momentum, qubits, and gates for optimal values, as described in Section 3.2.

## 5. Discussion

**Figure 12:**
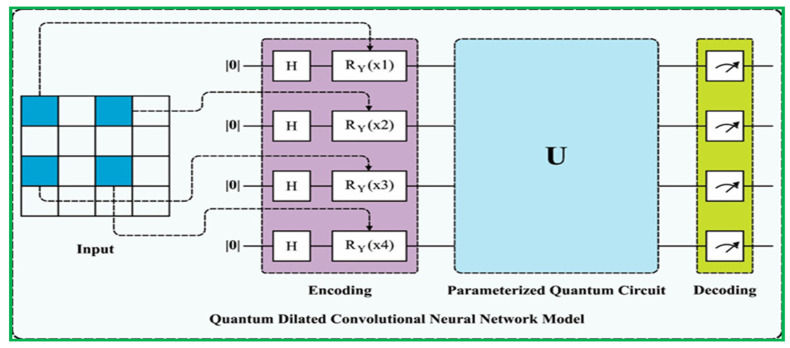
Integration of dilated inputs into the quanvolutional layer as a quantum circuit to perform operations differently than a conventional convolutional layer.

The topic of discussion is the QCNN model. The conventional QCNN model served as the benchmark model for our study, utilized initially as the first quanvolutional layer, as depicted in Figure 2 and Figure 12. The QCNN model shares the same structure as our QDCNN model, with the sole distinction being the utilization of a conventional quantum kernel instead of a dilated quantum kernel, which we implemented after six dilated convolutions, as shown in Figure 2. In the up-sampling part of the network, we employed QDCNN. Each model has a random quantum circuit composed of two layers, each containing four qubits. These layers consist of four parameters, which can be either non-trainable or trainable. It is imperative that all these circuits with random characteristics possess an equal architectural framework, which is established through the utilization of a uniform random seed.

The resulting state of the 4-qubit system undergoes additional transformation through a subsequent random parameterized quantum circuit, potentially generating entanglement. The decoding method employed in this study was similar to the approach described in reference [79]. In this approach, each expectation value is assigned to a distinct channel inside a single output pixel. Consequently, despite the presence of a single filter, the quantum layer has the ability to convert the input two-dimensional image into four distinct feature maps. The inclusion of a quantum layer in the model may enhance its performance by enabling correlation among the channels of the output feature maps. The QDC layer in the Massachusetts road datasets is responsible for extracting a feature tensor of size 500 × 500 × 32 from the input image, which has dimensions of 500 × 500. This feature tensor is then passed through the rest of the network shown in Figure 2, resulting in two output probabilities: either road pixels or non-road pixels. To assess the influence of the dilation rate on the performance of the model, we examined QDCNN models with a dilation rate of 2, as depicted in Figure 12.

The comparative analysis is based on various fundamental parameters used in the evaluation of road extraction, including IoU, recall, precision, and F1 score. The results outperformed other state-of-the-art methods, as shown in Figure 8 and Figure 9. The quantitative results are presented in Table 4, Table 5 and Table 6.

**Figure 13:**
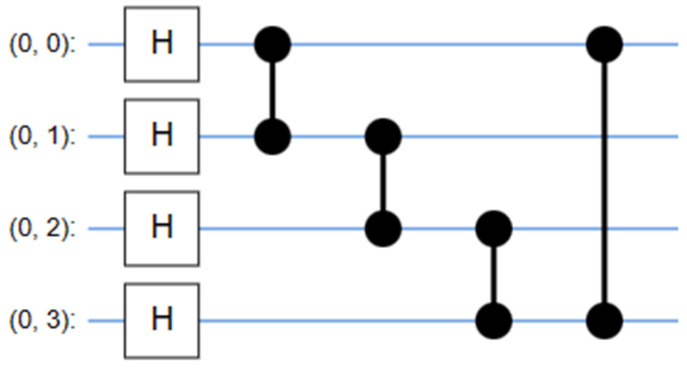
Quantum circuit.

Within the domain of remote sensing and semantic segmentation, the emergence of quantum computing signifies a fundamental transformation that has the capacity to profoundly alter the manner in which we extract valuable insights from Earth observation data. Conventional approaches, which heavily rely on traditional computing systems, frequently encounter difficulties in accurately capturing intricate details and contextual subtleties in remote sensing images, specifically in the context of road extraction. Quantum convolutional neural networks (QDCNNs) coupled with Archimedes optimization techniques present a viable avenue for future research and development. QDCNNs have the ability to improve the accuracy and efficiency of road extraction in remote sensing (RS) imagery by utilizing the parallelism and computational capabilities of quantum bits (qubits) in quantum circuits, as shown in Figure 13. This allows for the simultaneous processing of numerous locations of interest. The utilization of quantum-based methodologies exhibits the potential to greatly propel the domain of remote sensing, facilitating enhanced accuracy and expedited mapping of roads and infrastructure. This is particularly crucial for various applications such as urban planning, disaster management, and environmental monitoring.

Nevertheless, it is crucial to recognize that the utilization of quantum computing for image analysis remains a developing domain fraught with numerous technological obstacles. The successful deployment of quantum-dilated convolutional neural networks (QDCNNRE) is contingent upon the accessibility of quantum hardware that possesses a growing quantity of qubits and enhanced error rates. In addition, the ongoing research efforts are focused on the development of quantum algorithms that are specifically designed for the task of semantic segmentation. With the maturation of quantum technology and the increasing synergy between quantum and classical computing, significant breakthroughs in road extraction and other remote sensing applications are expected. These advancements have the potential to contribute to a more sustainable world that is informed by data.

## 6. Conclusions

This research article introduces the novel ATP-QDCNNRE method, aiming to enhance the efficiency and accuracy of road extraction from remote sensing images. Our methodology combines a synergistic integration of deep learning techniques and quantum dilated convolutional neural networks (QDCNN), inspired by principles derived from quantum computing. We employed the QCNN model, which shares a similar structure with the QDCNN model but distinguishes itself by incorporating dilated quantum kernels.

The utilization of quantum kernels significantly enhanced the model’s ability to effectively capture complex local and global contextual information. All experiments maintained uniform parameter initialization approaches and optimizers throughout the training process. The training procedure involved converting unprocessed imagery into feature maps using non-trainable quantum filters. Each model underwent training for 50 epochs, with a mini-batch size of 32. We employed the Adam optimizer with a learning rate of 0.01. To handle the computational requirements associated with training parametric quantum circuits within the trainable quantum filter, we reduced the batch size to four. For implementation, we utilized the PennyLane library and PyTorch. PennyLane is a Python-based open-source platform that facilitates hybrid quantum-classical computations by providing automatic differentiation support. Considering the significant quantum circuit executions in the parameter-shift rule scheme, we chose to train all hybrid models using the default.qubit built-in simulator in PennyLane. This simulator’s PyTorch interface supports backpropagation. The experiments were conducted on a Windows operating system using Intel Xeon CPUs and NVIDIA GRID RTX8000-12Q GPUs, ensuring optimal computational efficiency. The experimental results obtained from analyzing the Massachusetts road dataset provide evidence supporting the effectiveness of the ATP-QDCNNRE method.

The proposed approach outperformed recent methodologies, as evidenced by its superior performance on multiple evaluation metrics. These metrics included an intersection over union (IoU) of 75.28%, mean intersection over union (MIoU) of 95.19%, F1 score of 90.85%, precision of 87.54%, and recall of 94.41%. The optimization of parameters in quantum circuits was achieved using various approaches, such as the variation in quantum Eigensolver (VQE) and the quantum approximate optimization process (QAOA), which integrated the Archimedes optimization process. In the domain of hyperparameters for quantum dilated convolutional neural networks (CNNs) used in the context of semantic segmentation, we conducted a comprehensive optimization process for key parameters, including learning rate, mini-batch size, momentum, optimizer selection, and weight decay. These factors significantly impacted both the model’s performance and the speed of training. The ATP-QDCNNRE method represents a novel advancement in utilizing quantum-computing-inspired deep learning techniques for road extraction from RS imagery. However, there are certain computational constraints associated with significant processing resources required for quantum computing and hyperparameter optimization. These limitations could potentially impact the feasibility of implementing our methodology for real-time road extraction tasks or large-scale datasets. Nevertheless, the ATP-QDCNNRE method marks significant progress in the field of road extraction from remote sensing data.

The results revealed from the ATP-QDCNNRE technique present intriguing possibilities for future research in this field. The potential of quantum computing to improve the efficiency of road extraction tasks is highlighted by the model’s effective performance, as evidenced by its visual and quantitative findings on the Massachusetts road dataset. This finding presents opportunities for investigating the utilization of quantum integration with conventional CNN algorithms in the fields of remote sensing and image analysis. Such exploration has the potential to result in notable progress in the processing of geospatial data and the management of infrastructure. Subsequent investigations may be directed towards further enhancing the model, optimizing its architectural design, and investigating its applicability in many real-world contexts.

## Figures and Tables

**Table 3:** Hyperparameters in QDCNNRE.

Hyperparameter	Description
Quantum circuit depth	The depth of quantum circuits or layers in the network (3 quantum layers).
Quantum gate parameters	Parameters specific to quantum gates used in the model. Example: Gate time and type (e.g., CNOT, Hadamard).
Momentum qml.optimize. ArchimedesOptimizer	Momentum is a mathematical optimization method used to enhance convergence speed and stability during the training of machine learning models. The “qml.optimize. ArchimedesOptimizer” is a quantum optimization algorithm used for solving optimization problems on quantum devices.
Loss Function	The choice of loss function for semantic segmentation is cross-entropy loss.
Activation function	The type of activation function used in the network. ReLU and Sigmoid.
Learning rate	Controls the step size during optimization.

**Table 4:** Comparative outcomes of ATP-QDCNNRE approach with DL techniques on the Massachusetts road dataset.

Model	IoU	MIoU	F1 Score	Precision	Recall	FPS
PSPNet [75]	58.91	72.23	75.22	74.37	76.09	75
D-LinkNet [76]	61.45	75.72	80.61	78.77	82.53	96
LinkNet34 [77]	61.35	75.87	80.17	78.77	81.63	105
CoANet [78]	61.67	76.42	81.56	78.53	84.85	61
CoANet-UB [78]	64.96	80.92	88.67	85.37	92.24	40
MECA-Net [64]	65.15	82.32	78.90	78.39	79.41	89
ATP-QDCNNRE (Ours)	75.28	95.19	90.85	87.54	94.41	158

**Table 5:** Ablation experiment.

Methods	Precision (%)	Recall (%)	F1-Score (%)	IoU (%)
Baseline (DCNN)	58.47	76.10	66.10	65.67
DCNN + (Quanvolutional) QDCNNRE	80.07	72.22	76.40	72.04
Baseline + ATP-QDCNNRE	87.54	94.41	90.85	75.28

**Table 6:** FPS analysis of the ATP-QDCNNRE approach with other methodology on various road datasets.

Methods	FPS on Various Road Dataset
PSPNet [76]	75
D-LinkNet [77]	96
LinkNet34 [78]	105
CoANet [79]	61
CoANet-UB [79]	40
MECA-Net 80]	89
PSPNet [76]	75
ATP-QDCNNRE (Ours)	158

## Data Availability

Open source data were used and the data will be made available upon request.
